# Baseline Gait and Motor Function Predict Long-Term Severity of Neurological Outcomes of Viral Infection

**DOI:** 10.3390/ijms24032843

**Published:** 2023-02-02

**Authors:** Moumita Karmakar, Aracely A. Pérez Gómez, Raymond J. Carroll, Koedi S. Lawley, Katia A. Z. Amstalden, C. Jane Welsh, David W. Threadgill, Candice Brinkmeyer-Langford

**Affiliations:** 1 Department of Statistics, College of Science, Texas A & M University, College Station, TX 77843, USA; 2 Department of Veterinary Integrative Biosciences, School of Veterinary Medicine and Biomedical Sciences, Texas A & M University, College Station, TX 77843, USA; 3 Department of Molecular and Cellular Medicine, Texas A & M Health Science Center, Texas A & M University, College Station, TX 77843, USA

**Keywords:** Collaborative Cross, TMEV, gait, DigiGait, QTL

## Abstract

Neurological dysfunction following viral infection varies among individuals, largely due to differences in their genetic backgrounds. Gait patterns, which can be evaluated using measures of coordination, balance, posture, muscle function, step-to-step variability, and other factors, are also influenced by genetic background. Accordingly, to some extent gait can be characteristic of an individual, even prior to changes in neurological function. Because neuromuscular aspects of gait are under a certain degree of genetic control, the hypothesis tested was that gait parameters could be predictive of neuromuscular dysfunction following viral infection. The Collaborative Cross (CC) mouse resource was utilized to model genetically diverse populations and the DigiGait treadmill system used to provide quantitative and objective measurements of 131 gait parameters in 142 mice from 23 CC and SJL/J strains. DigiGait measurements were taken prior to infection with the neurotropic virus Theiler’s Murine Encephalomyelitis Virus (TMEV). Neurological phenotypes were recorded over 90 days post-infection (d.p.i.), and the cumulative frequency of the observation of these phenotypes was statistically associated with discrete baseline DigiGait measurements. These associations represented spatial and postural aspects of gait influenced by the 90 d.p.i. phenotype score. Furthermore, associations were found between these gait parameters with sex and outcomes considered to show resistance, resilience, or susceptibility to severe neurological symptoms after long-term infection. For example, higher pre-infection measurement values for the Paw Drag parameter corresponded with greater disease severity at 90 d.p.i. Quantitative trait loci significantly associated with these DigiGait parameters revealed potential relationships between 28 differentially expressed genes (DEGs) and different aspects of gait influenced by viral infection. Thus, these potential candidate genes and genetic variations may be predictive of long-term neurological dysfunction. Overall, these findings demonstrate the predictive/prognostic value of quantitative and objective pre-infection DigiGait measurements for viral-induced neuromuscular dysfunction.

## 1. Introduction

An individual’s gait—the speed and length of the steps, the degree to which consecutive steps overlap, the angle of each foot, etc.—can be affected by various neurodegenerative conditions. Several of these conditions can be influenced or precipitated by viral infections such as Parkinson’s disease (PD) [1,2,3,4,5,6,7,8], amyotrophic lateral sclerosis (ALS) [9,10,11], and multiple sclerosis (MS) [12,13,14,15,16,17,18,19,20]. These diseases can have characteristic gait patterns [21], such as the shortened, shuffling gait seen in PD [22,23,24]. However, knowledge of the gait pattern characteristics of different diseases has often come from comparisons with healthy controls rather than longitudinal data from the same individual. Because disease-related gait changes occur after disease onset, it is difficult to discern which differences were already present while the individual was still healthy. Moreover, neurological symptoms such as gait changes can arise gradually over time that, in some cases, can stretch over a period of years.

Owing to the dearth of pre-diagnosis gait data in humans, animal models are logical alternatives for identifying which aspects of gait can have the most prognostic value. In the current study, mice with human-relevant genetic diversity were evaluated via a computerized treadmill (DigiGait) to determine whether certain pre-infection gait characteristics could be associated with greater long-term disease severity in neurodegenerative diseases resulting from viral infection. DigiGait provides objective gait measurements for rodents which are analogous to measurements used for evaluating gait in humans affected by diseases such as PD [25,26]. These aspects or parameters of gait do not necessarily have any pathologic influences on their own but could indicate morphometric differences reflecting pre-existing susceptibilities. In humans, such baseline measurements could be useful for developing patient-specific treatment plans following viral infections that affect neurological function.

A well-known mouse model of multiple sclerosis, known as Theiler’s murine encephalomyelitis virus (TMEV)-induced demyelinating disease, was utilized to model human neurodegenerative outcomes in response to viral infection. For several decades, TMEV infection in various inbred mouse strains, especially SJL/J, has been studied as a viral model of MS. More recently, the roles of genetic diversity in TMEV-induced neurological outcomes have been evaluated. By infecting recombinant-inbred mice of the Collaborative Cross (CC) resource, we found that TMEV can cause symptoms similar to PD and ALS, along with MS and other neurodegenerative human diseases. These CC mice were the result of 20+ generations of crossbreeding eight inbred (“founder”) mouse strains. After no more recombination could be detected in the offspring, each distinct line of CC mice continued to be propagated by inbreeding, resulting in many genetically distinct CC mouse strains that are both inbred and highly diverse, capturing 90% of all the variation found in the original eight founder mouse strains [27,28,29]. Therefore, it is reasonable to view CC mouse strains as reflecting different human populations that are genetically distinct from each other, each having maximum genetic diversity contributing to highly divergent outcomes to the same event (such as infection with the same virus).

Previously, our laboratory established a phenotyping pipeline to categorize the neurological outcomes of CC mice infected with TMEV [30,31]. Limb weakness, balance, coordination, and paralysis among other clinical signs were evaluated. Gait changes have been previously shown to correspond to disease outcomes during TMEV [31,32,33]; therefore, the objective quantitative DigiGait treadmill platform (Mouse Specifics, Boston, MA, USA) was employed to capture outcomes not easily noticed by the human eye. The applicability of DigiGait has been demonstrated in rodent models of human conditions such as ALS and PD (e.g., [34,35,36,37]). Accordingly, in this study, gait parameters measured by DigiGait were associated with long-term neurological disease severity in different CC mouse strains. Furthermore, associations were found between genomic regions and some of these measurements, thereby identifying parallels between the mouse gait parameters correlated with disease severity and human gait characteristics.

## 2. Results

### 2.1. DigiGait Parameters Provided Objective Measures of Gait and Motor Function

The hypothesis for this study was that pre-infection gait characteristics could predict long-term neurological symptoms following a viral infection. DigiGait parameter measurements quantify multiple aspects of gait and motor function that could be affected differently by challenges to motor neuron function, such as TMEV infection. Therefore, quantitative and objective pre-infection DigiGait measurements with predictive or prognostic capabilities were sought.

Previous work by the authors described post-infection DigiGait measurements taken at the juncture between acute and chronic infection (21 days post-infection [d.p.i.]), and just prior to sacrifice (90 d.p.i.) [31]. This work demonstrated the utility of DigiGait for discerning between subsets of mouse strains with bradykinetic gait, which tended to have Parkinsonian-type phenotypes following TMEV infection, as opposed to mice with other gait profiles that exhibited relatively normal phenotype profiles following TMEV infection. For the current study, however, pre-infection, baseline (0 d.p.i.) DigiGait measurements were used. To normalize inherent differences in body size between mouse strains, the weight of each mouse at the time of assessment was included in the DigiGait algorithm. In this way, gait profiles were developed showing the natural strain differences prior to the introduction of the viral infection (Table S1).

After this initial gait assessment, the mice were infected with TMEV as previously reported [38,39], and their neurological phenotypes were recorded daily throughout the acute phase (first two weeks post-infection), and weekly thereafter up to 90 d.p.i. Sham-infected control mice were included for every strain and both sexes, and all phenotypes were evaluated relative to those of the relevant control mice. The more often a given phenotype (paresis, paralysis, piloerection, kyphosis, seizures, limb clasping, and delayed righting reflex) was observed, the higher the cumulative score was for that phenotype at the end of the study. Paralysis and paresis were scored for each limb individually and therefore had a larger effect on the overall cumulative score. The “90 d.p.i. phenotype score” accounted for all phenotypes [31], and because many phenotypes were somewhat interdependent (for example, paralysis would impede righting reflex), this overall score accurately reflected the differences in severity of disease expression between each strain. Indeed, it was found that this scoring system reliably tracks TMEV-induced central nervous system (CNS) damage [38]. For this study, previously obtained 90 d.p.i. phenotype scores were considered as the endpoints for the prognostic analysis of using pre-infection DigiGait measurement values.

### 2.2. Pre-Infection DigiGait Measurements Were Significantly Associated with 90 d.p.i. Phenotype Scores

To determine whether gait patterns may serve as prognostic markers for viral-induced motor alterations, of the 131 pre-infection DigiGait measurement values, stepwise regression identified those with the best predictive value and the most significant association with the 90 d.p.i. phenotype scores. Briefly, stepwise regression involves fitting multiple regression models to identify the optimal combination of “variables” (e.g., DigiGait parameters and 90 d.p.i. scores) and has been found particularly useful when using data from complex populations [40,41]. For this study, stepwise regression not only was used to identify significant associations, but also was used for validating these associations, confirming the predictive value of the associated measurements.

The final model determined using “step Akaike Information Criterion (AIC)” yielded a *p*-value of zero, demonstrating the overall significance of the selected model describing the association between the selected pre-infection variables (DigiGait parameters) and the 90 d.p.i. phenotype score. Between five and ten DigiGait parameters per limb were identified as being statistically associated with the 90 d.p.i. phenotype score at the 5% significance level. Thus, a total of 31 parameters for the four limbs were identified as significant (out of 131 considered) (Table 1). Of the 31 significant parameters, % Propel Stance, Brake, MAX dA/dT (maximum paw area over time), Midline Distance, and Paw Drag were significantly associated to two separate limbs. In addition, Paw Placement Positioning (PPP), Stride, and Stride Frequency were each significantly associated to three separate limbs, with hindlimb parameters accounting for 19 of the 31 parameters.

Next, a linear mixed model was fitted to reveal how the genetic variability from the CC strains affected the association between 90 d.p.i. phenotype scores and DigiGait variables. This procedure was repeated separately for each limb. In the linear mixed model, each strain was used as the random effect and significant DigiGait variables, and those identified by stepwise regression were treated as fixed effects. Following the linear mixed model assessment, strain-inherent gait patterns and characteristics were, on their own, not sufficient to explain the significant associations between DigiGait parameters and 90 d.p.i. phenotype scores (Table 1). In other words, regardless of mouse strain, the associations identified between pre-infection DigiGait measurements and 90 d.p.i. phenotype scores were relevant. This increases the prognostic value of these DigiGait measurements: these measurements are not only useful for certain CC strains, or certain 90 d.p.i. scores, but can be utilized for predictive purposes in future studies using any mouse strain.

Each DigiGait parameter was previously classified based on the aspect of gait it measured [31,42,43,44]. Most, if not all, gait parameters are interrelated to some extent, but different pathologies may have a greater impact on certain aspects of gait. For example, patients with ALS, PD, and Huntington’s Disease exhibit differences in specific gait aspects, in part due to different CNS region involvement [23,45]. Therefore, the 31 variables significantly associated with the 90 d.p.i. phenotype score were categorized based on relevance to the spatial, temporal, or postural properties of gait or intraindividual variability [46]. Briefly, temporal gait parameters include aspects of gait measured in units of time (e.g., seconds [s]), spatial gait parameters include aspects of gait measured in units of length/size (e.g., centimeters [cm]), parameters classified as “intraindividual variability” reflect step-to-step variances measured in degrees or percentages, and postural gait parameters include aspects of gait pertaining to how much each step deviates from some standard, such as centroid axis (Axis Distance) or transverse midline (Midline Distance), or step-to-step paw placement (Paw Area Variability, Paw Angle, and Paw Drag).

Previously, temporal aspects of gait were measured by approximately 38% of all DigiGait parameters, spatial and postural aspects were captured by 25% and 19%, respectively, and roughly 15% measured intraindividual variability [31]. The categories of the 31 significant variables identified using the stepwise regression model, when weighted by the number of limbs for each variable found as significant, were represented at slightly different percentages: 39% temporal, 32% spatial, 23% postural, and 6% intra-individual variability. In other words, there was a noticeable increase in the representation of DigiGait parameters measuring spatial and postural aspects of gait in this study.

**Table 1:** Pre-infection (baseline) DigiGait parameters for each limb (second column from left) were significantly associated with 90 d.p.i. phenotype scores via stepwise regression analysis. These parameters represent different aspects of gait, listed in the third column from the left. ^#^ Parameters significantly associated with TMEV response categories via one-way ANOVA (as shown in Figure 1) are indicated with *p*-values listed in the rightmost column. * Indicates parameters for which significant QTL were identified (QTL are listed in Table 2).

Limb	DigiGait Parameter	Parameter Category (from [46])	Association with Response Categories (from [47]) and/or Sex
Left forelimb (LF)	Swing *	temporal	
	Brake ^#^*	temporal	*p* = 0.0413
	Stance	temporal	
	Stride *	spatial	
	MAXdA/dT	temporal	
Left hindlimb (LH)	Stride ^#^	spatial	*p* = 0.00573 (female resilient vs. female susceptible: *p* = 0.0064; female resilient vs. male susceptible: *p* = 0.0188)
	% Propel Stance	temporal	
	Stride Frequency ^#^	spatial	*p* = 0.0375 (female resilient vs. female susceptible: *p* = 0.032; female resilient vs. male susceptible: *p* = 0.0034)
	Stance Width Coefficient of Variance (CV) ^#^	intraindividual variability	*p* = 0.0169 (female resilient vs. male resistant: *p* = 0.0396; female resilient vs. male susceptible: *p* = 0.0077)
	Paw Placement Positioning	spatial	
	Paw Drag ^#^	postural	*p* = 0.00425
	Hind Limb Shared Stance Time ^#^*	temporal	*p* = 0.0287
Right forelimb (RF)	Brake *	temporal	
	Propel ^#^	temporal	*p* = 0.0222
	Stride ^#^	spatial	*p* = 0.0373
	Stride Frequency *	spatial	
	Paw Area at Peak Stance	postural	
	MAXdA/dT	temporal	
	Paw Placement Positioning	spatial	
	Midline Distance	postural	
Right hindlimb (RH)	% Propel Stance *	temporal	
	% Propel Stride	temporal	
	Stance/Swing *	temporal	
	Stride Frequency	spatial	
	Paw Angle	postural	
	Paw Angle Variability	intraindividual variability	
	Overlap Distance	spatial	
	Paw Placement Positioning	spatial	
	Midline Distance	postural	
	Axis Distance	postural	
	Paw Drag ^#^*	postural	*p* = 0.0242

**Table 2:** DigiGait parameters with statistically significant QTL are listed in chromosome order. QTL chromosomal positions are shown in the third column from the left. The program used for QTL mapping (gQTL, [48]) uses data from an SNP assay which includes SNPs with different names than SNPs of the Mouse Phenome Database (MPD), which uses Reference SNP Identification (rsID). Since gQTL and MPD use different names for the same SNPs, both names were listed for each SNP in the second and third columns. The column labeled “*p*-value” gives the *p*-value for each QTL. The “Location” column provides the chromosome and base pair (chr:bp) location of each SNP. The column labeled “Prox-Dist (Mb)” shows Mb positions for the proximal and distal boundaries of each QTL region, and to the right, the numbers of genes (including pseudogenes) located in each region are listed. The column “Significant DEGs in region” gives the number of differentially expressed genes (DEGs) with statistically significant expression between infected and sham-infected mice of the same sex and strain; the final column on the right lists the number of significant DEGs which are known protein-coding genes. (LF = left forelimb; LH = left hindlimb; RF = right forelimb; RH = right hindlimb).

Phenotype	Sex	SNP ID (gQTL)	rsID (MPD)	*p*-Value	Location (chr:bp)	Prox-Dist (Mb)	Number of Genes in Region	Significant DEGs in Region	Protein-Coding DEGs
Swing LF	both	UNC23682331	rs30743246	1.59 × 10^−6^	14:21992279	9.17–25.50	376	7	0
Brake LF	both	UNC27374397	rs4217379	2.03 × 10^−6^	16:90390967	86.46–90.56	100	1	0
Stride LF	both	UNC855090	rs51387167	9.18 × 10^−9^	1:67861707	64.57–67.96	400	4	3
Hindlimb Shared Stance Time LH	both	UNC30823189	rs30323288	2.41 × 10^−7^	X:51777164	50.67–51.84	687	5	0
Brake RF	F	JAX00139829	rs29969310	1.40 × 10^−10^	6:36890310	34.85–36.99	53	1	1
Stride Frequency RF	both	UNC19870031	rs224655440	2.18 × 10^−6^	11:69589978	64.57–67.96	942	18	13
% Propel Stance RH	F	UNC26076456	rs30829258	9.86 × 10^−8^	15:89682500	89.59–91.33	70	2	2
Stance/Swing RH	M	UNC16337966	rs37517810	2.34 × 10^−7^	9:49759868	49.70–52.20	950	12	9
Paw Drag RH	F	UNC30588773	rs50371642	7.41 × 10^−8^	19:59415570	59.05–61.26	57	0	0

**Figure 1:**
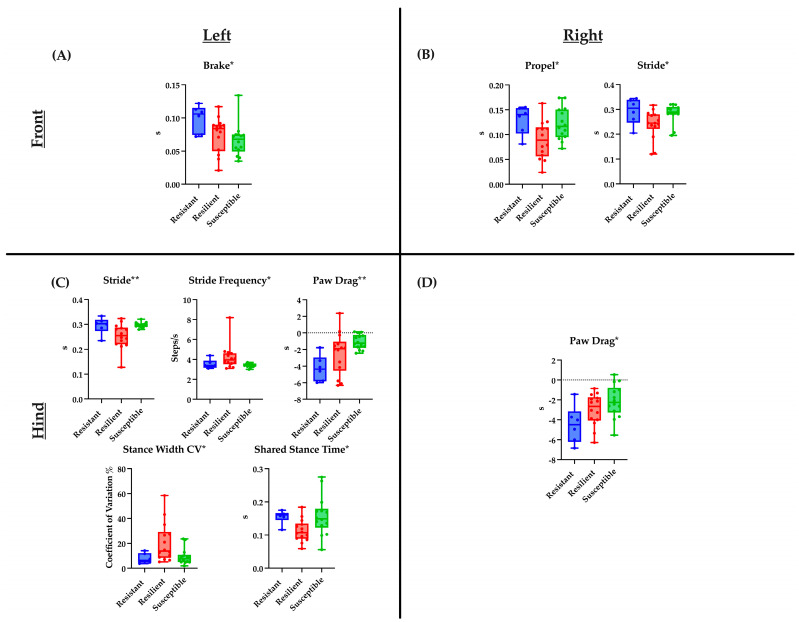
Baseline DigiGait parameters significantly associated with the three TMEV response categories are shown. Plots describe the distribution of measurement values for mice belonging to each of the three categories (resistant, resilient, and susceptible), with horizontal bars depicting the average values within each category. Each panel indicates parameters for each limb ((**A**)—front left limb, (**B**)—front right limb, (**C**)—hind left limb, and (**D**)—hind right limb). *p*-values were determined using a one-way ANOVA (see Section 4 Materials and Methods); * *p* < 0.05, ** *p* < 0.01, s: seconds.

### 2.3. Relationships between Strain Categories and Baseline DigiGait Measurements

Previously, CC strains were categorized based on their TMEV disease course (“response category”), with strains designated as resistant, resilient, or susceptible, based on the level of TMEV RNA persistence and severity of neurological phenotypes [47]. These categories reflect that TMEV RNA levels do not consistently correlate with disease severity as represented by the 90 d.p.i. phenotype score. Resistant and resilient strains had lower 90 d.p.i. phenotype scores, and resilient and susceptible strains had measurable levels of TMEV RNA at 90 d.p.i. DigiGait measurement values were analyzed in respect to the response groups to determine whether pre-infection gait patterns could predict whether a given strain would be classified in any one of the response groups. Among the pre-infection DigiGait parameters listed in Table 1, nine significant associations between measurement values and response categories were identified, including one (left hindlimb Stance Width CV) for which measurement values were also significantly associated with sex. The measurement values for the mice in each of the three response categories provided more context for interpreting the connection between gait patterns and phenotype scores (Figure 1). For example, mice with the highest baseline measurement values for left and right hindlimb Paw Drag tended to experience the greatest disease severity, falling under the “susceptible” response category following TMEV infection.

Additionally, the influence of sex on the relationship between TMEV response categories and pre-infection DigiGait measurements were evaluated. There were fewer individuals per category, yet statistically significant differences were identified between females and males, and between females from different TMEV response groups. These differences were found in three parameters, all measured in the hindlimbs. A significant difference was found in left hindlimb Stride measurements for females in the resistant vs. resilient categories, and for females of the resilient vs. susceptible categories. Furthermore, Stride measurements were significantly different between resilient females and susceptible males. A related parameter, left hindlimb Stride Frequency, also showed significant differences between resilient and susceptible females, as well as between resilient females and susceptible males. Additionally, the left hindlimb Stance Width CV was significantly different between resilient females and both resistant and susceptible males. Finally, right hindlimb Axis Distance measurements were significantly different between resilient females and susceptible males. Paw Drag values for both hindlimbs tended to be greatest in susceptible mice. Although this finding was not statistically significant, its potential biological relevance was noteworthy. Regardless of sex, mice with higher Paw Drag values prior to viral infection were classified as the most severely affected of all mice by 90 d.p.i. Overall, DigiGait parameters, associated per limb, have been identified according to their TMEV response categories narrowing the list from the by identified 31 parameters originally found. These nine parameters of interest may allow for future studies to focus on the temporal motor degradations observed in viral-induced neurological mouse models.

### 2.4. Quantitative Trait Loci (QTL) Mapping

QTL mapping was performed for pre-infection DigiGait measurements to identify loci potentially involved in strain-inherent gait differences. Nine significant QTL for pre-infection DigiGait parameters were associated with 90 d.p.i. phenotype score. Four of these QTL were sex specific (three female-only and one male-only), indicating that their effects were not as strong in all mice combined as when in one sex or the other.

Significant QTL are listed in Table 2. Manhattan plots, founder genotypes, and gene identities located within QTL regions are provided in Figures S2 and S3 and Table S2.

Inheritance played a major role in the Collaborative Cross mice as certain founder strains were found to have a stronger influence on the gait measurements of the resistant, resilient, and/or susceptible strains. For resilient strains, several measurements were significantly associated with QTL inherited from the wild-derived founders of the CC: CAST/EiJ, PWK/PhJ, and WSB/EiJ (Table 2 and Figure S3; [49,50]). For example, a QTL for left forelimb stride was inherited from CAST/EiJ in the resilient strain CC015 and WSB/EiJ in CC043. Resilient CC037 mice inherited the QTL for left hindlimb shared stance time from the WSB/EiJ founder strain. Another resilient strain, CC027, inherited the QTL for right forelimb stride frequency from WSB/EiJ; however, this was also true for the resistant strain CC036. For each of the nine significant QTL listed in Table 2, up to three resilient strains inherited genotypes from a wild-derived strain (Figure S3) [49,50]. By contrast, resistant and susceptible strains inherited wild-derived genotypes for 5 and 2 QTL, respectively. Therefore, the gait of resilient strains were often influenced by genetic inheritance from wild-derived founder strains, although not consistently for all resilient strains or exclusively for any one parameter.

Next, the expression of genes in the QTL regions (Table S2) was investigated to identify correlations between gene expression and the nine gait parameters listed in Table 2. Gene expression levels were previously evaluated via RNAseq for the hippocampus and thoracic spinal cord at 90 d.p.i. [47]. These data were originally collected for both TMEV-infected and sham-infected mice of both sexes and all strains to ascertain differences in the response of each strain to TMEV infection. Of the 3635 genes and pseudogenes located within QTL regions, 51 were expressed at significantly different levels between infected and sham-infected mice of the same sex and strain; 23 of these differentially expressed genes (DEGs) were classified as non-coding RNA, pseudogenes, or predicted genes. The ontologies of the remaining 28 known protein-coding DEGs were relevant to different aspects of gait and/or viral infection. As expected, there was no 1-to-1 correspondence between the DEG expression level and phenotype measurement value; however, the influence of these genes was likely contextual: each DEG affected some facet(s) of different gait parameters, with multiple DEGs collectively contributing to the phenotype. Thus, genetic markers associated with gait and viral infection have been identified that can serve as potential markers of interest in prognostic gait studies.

## 3. Discussion

Gait changes are common to many neurological conditions, but oftentimes these changes are noted in relation to control samples rather than longitudinal measurements of the same individual. Mouse model systems provide the ability to measure gait prior to disease development, so that it is possible to identify relevant aspects of gait which can indicate the likelihood of an individual mouse experiencing a more or less severe expression of a disease compared to other mice. The DigiGait system offers quantitative and objective measurements of numerous gait patterns in murine models. This system provides a practical and functional method to assess gait changes as part of a disease process, compared to other gait analysis procedures. For instance, DigiGait was more sensitive than other methods, such as CatWalk, when measuring gait impairment resembling MS in experimental autoimmune encephalitis mice [51]. Similar to our study, the system was used to note significant changes in spatial and temporal gait patterns of Ross River virus (RRV) that disseminates in skeletal muscles and joints [52]. One evident limitation of our study was that while the gait of a quadruped murine model may not directly match to the gait of human bipeds, there have been documented gait development aspects that mirrored those to humans [46]. While humans and mice do not share entirely similar gait patterns, similarities in gait aspects—such as whether it is affected by a lack of strength, coordination, etc.—support the potential for identifying prognostic gait patterns in humans. In addition, the DigiGait system accounts for the standardization of gait speed and mouse weight to directly extrapolate proper gait assessments. Thus, the DigiGait measurement values may provide valuable context for interpreting the connection between murine gait patterns and gait disturbances observed in human neurological disorders.

The current study demonstrates how pre-existing differences in gait can predict possible neurological dysfunction resulting from long-term viral infection, as measured by the collective 90 d.p.i. phenotype score. Of the 131 discrete parameters measured at the pre-infection time point (“baseline”; Table S1), 20 demonstrated a statistically significant relationship with the score. These gait parameters represent multiple aspects of gait (e.g., temporal, spatial, postural, and intraindividual variability). The parameters significantly associated with the 90 d.p.i. phenotype score tended to bias more toward the spatial and postural aspects of gait than expected, such as stride and paw drag. This suggests that the inherent body conformation of a mouse is predictive of the severity of long-term physical effects following TMEV infection. Moreover, eight of the 20 parameters were significant for more than one limb. These eight parameters included Paw Placement Positioning, Stride, and Stride Frequency, which represent spatial aspects of gait and were associated with 90 d.p.i. phenotype scores for three limbs each. % Propel Stance, Brake, MAX dA/dT, Midline Distance, and Paw Drag, which measure temporal and postural aspects of gait, were significant for two limbs each.

Gait parameters significantly associated with 90 d.p.i. phenotype score for multiple limbs were likely strain-dependent and robust in relation to body size. For example, Paw Placement Positioning is measured in centimeters and reflects the extent of overlap between the fore and hind paws on the same side of the body. If the forelimb and hindlimb stances are roughly equal, the value of this parameter is close to zero. The Stride parameter indicates the duration of one complete stride for one paw, which depends on both the speed of motion and stride frequency [53]. Meanwhile, the Stride Frequency parameter measures the number of times a paw takes a complete stride per second. Therefore, Stride Frequency reflects the tempo of the gait [53,54]. All three of these parameters describe aspects of balance and rhythmicity of gait, and while they are all considered “spatial” aspects of gait because their measurements are calculated by DigiGait software from real digital footprints [46], there is also a temporal connotation to Stride and Stride Frequency. The overlap of spatial and temporal gaits have also been described in humans and specifically in the elderly and in stroke patients [25,26,55]. Consistent coordinated spatial and temporal features of gait result in steady locomotor patterns, which may be disrupted as a result of injuries and diseases affecting the nervous system (e.g., Parkinson’s disease; [56]).

Pathological circumstances can also affect the % Propel Stance, Brake, MAX dA/dT, Midline Distance, and Paw Drag, which measure temporal and postural aspects of gait and were significant for two limbs each. For example, measurement values for % Propel Stance indicate the percentage of the stance phase in which the paw comes off the treadmill belt to propel the mouse forward. Paw Drag measurements reflect the robustness of the lift-off stage of the paw from the treadmill belt. The 90 d.p.i. score was associated with % Propel Stance and Paw Drag values for both hindlimbs, but not with forelimbs, which makes sense in the context of TMEV infection pathology: weakness and paralysis tend to be more prevalent in the hindlimbs [30,31]; thus, any pre-existing differences in these parameters would logically be amplified in infected mice. In contrast, forelimb measurements for the Brake and MAX dA/dT parameters were associated with 90 d.p.i. score. To maintain control and load distribution as needed, changes in hindlimb propulsion could be compensated by forelimb braking power and by adjusting MAX dA/dT, the maximal rate of change in paw area in contact with the treadmill belt during braking. Finally, Midline Distance measurements for both limbs on the right side of the body were associated with 90 d.p.i. score. Midline Distance values increased when forelimbs reached farther or hindlimbs extended farther. The relationship between Midline Distance and 90 d.p.i. score may be clarified when considering that TMEV infection occasionally results in weakness and/or paralysis on one side of the body only [31], leading to changes in balance and coordination that would be reflected in Midline Distance measurements. TMEV-induced lesions develop in different locations within the CNS depending on the mouse strain [57,58], and the effects of these lesions could amplify pre-existing gait tendencies for certain strains but not others. The 90 d.p.i. phenotype score provides a quantitative means of comparing the overall severity of neurological symptoms; however, considering this score in context with baseline gait parameters reveals a more holistic understanding of the physiological expression of the disease.

Along these same lines, 90 d.p.i. scores of different CC strains were evaluated in relation to how well each strain cleared the viral infection over 90 days. From this, three TMEV response categories were defined: “resistant”, “resilient”, and “susceptible” [47]. Resistant mice successfully cleared the infection while maintaining a relatively mild disease course, whereas resilient and susceptible mice did not clear the infection but diverged in symptom severity, with susceptible mice experiencing the most severe symptoms. Of the gait parameters with pre-infection measurements statistically associated with the 90 d.p.i. infection score, nine gait parameters were associated with response categories, five of which were specific to the left hindlimb (Table 1). Average measurement values for each of the nine parameters were distinctly different for the three response categories (Figure 1). For example, Paw Drag values increased from resistant to resilient to susceptible strains, suggesting that even before infection, the strains that would have a more susceptible outcome had a tendency to lift each hind paw less vigorously than the strains that would eventually be categorized as resilient or resistant. Similarly, pre-infection measurements of the Brake parameter were lowest in susceptible strains, indicating that these strains might have had less natural gait control as their steps had relatively less firm, full contact with the treadmill belt. However, other parameters did not have an obvious correlation with the TMEV response category, and often, the resilient strains had the highest or lowest values.

Resilient strains tended to have shorter stride times, greater stride frequencies, less time spent in the propel phase, and less hindlimb shared stance time (which typically increases with stride time [59]). These strains also had the highest values for Stance Width CV. Together, these pre-infection measurements suggest that resilient strains had quicker gaits than their resistant and susceptible counterparts. Prior research has demonstrated that natural gait and posture variations between strains can be attributed in part to genetic underpinnings and that wild-derived strains tend to have distinct gait patterns compared to those of many classical inbred strains [60].

Gait patterns do not usually differ by sex in healthy mice of the same strain [53], although neuropathologies can affect gait differently in females and males (for example, mouse models of Parkinson’s disease [61]). Therefore, it was not expected to identify sex differences in relation to pre-infection DigiGait parameters, which were measured before the viral infection. However, of the nine gait parameters associated with TMEV responses, four were also significantly different based on sex (Figure S1). The difference between resilient females and susceptible males was significant for all four parameters, and for two parameters there were significant differences between the response groups in females only. A significant difference was also found between the resilient females and resistant males. Every significant sex difference, in other words, involved the resilient female group. Resilient females had the shortest stride times, greatest stride frequencies, and greatest coefficient of variation in stance width (Stance Width CV) for the left hindlimb (a similar pattern was observed for the other limbs, but the effects were not significant). For the right hindlimb, resilient females showed the smallest deviation from the centroid axis (Axis Distance) of any group, and this was significantly different from susceptible males. Taken together, these findings suggest that resilient female mice had quicker, more symmetrical gaits than other mice prior to TMEV infection, which could indicate an improved ability to counteract gait deficits later on (for example, as seen for stance width variation in mice [62]). In general, however, the TMEV response category had a greater influence on gait measurements than sex, and it is unclear why only these four parameters showed substantial sex differences. It is intriguing to consider that resilient females, though unable to clear the viral infection by 90 d.p.i., could compensate from an ambulatory standpoint based on pre-existing advantages, e.g., slightly faster gait and greater balance.

## 4. Materials and Methods

### 4.1. Mice

Ethics statement: All procedures were approved by the Institutional Animal Care and Use Committee at Texas A&M University and performed under animal use protocol numbers 2017-0082 (approved 20 July 2017) and 2020-0065 (approved 21 May 2020). All experiments were performed in accordance with relevant guidelines and regulations. Mice were group-housed, and all testing performed during the light phase. Mouse strains, sexes, infection status, and 90 d.p.i. phenotype scores are shown in Table 3.

Previously, distinct TMEV “response categories” were identified in CC mouse strains [47]; the response categories were known for several strains included in the current study. “Resistant” strains had no or very little evidence of TMEV RNA, along with mild disease symptoms (low 90 d.p.i. phenotype scores) at 90 d.p.i. “Resilient” strains also had low 90 d.p.i. phenotype scores, but with high levels of TMEV RNA at 90 d.p.i. “Susceptible” strains had high 90 d.p.i. phenotype scores, indicating severe and persistent signs of the disease, along with high levels of TMEV RNA at 90 d.p.i.

### 4.2. DigiGait

DigiGait evaluations were performed prior to TMEV infection (0 d.p.i.) to establish the baseline gait profiles for each mouse and strain. The DigiGait rodent treadmill system provides quantitative and objective measurements of different aspects of gait, coordination, balance, and motor function. DigiGait has been useful for objectively assessing gait using identical contexts, such as treadmill speed, to minimize confounding influences in a timely manner [64]. The method provides quantitative data for each limb independently, and evaluates over thirty gait parameters per limb; thus, generating far more data points with greater accuracy than the traditional “foot print” method the authors have used in the past [30]. Importantly, the DigiGait apparatus considers the weight of each mouse when measuring these parameters; therefore, each mouse was weighed before walking on the treadmill. All mice walked on the DigiGait treadmill for a minimum of six consecutive steps per limb at the same speed (15 cm/s) and time of day. The treadmill belt was cleaned with 70% ethanol between each mouse performance. Measurements for each of the four limbs (left and right forelimbs and hindlimbs) were appraised independently using DigiGait and reported as separate values.

### 4.3. 90 d.p.i. Phenotype Score

**Figure 2:**
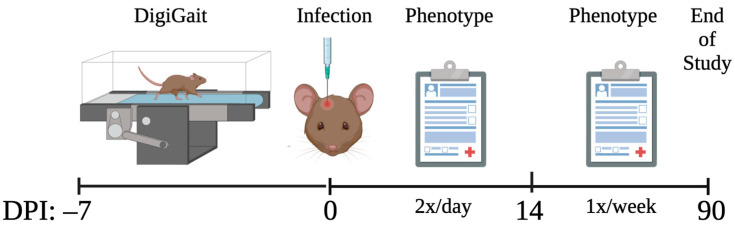
Prior to infection, different aspects of gait were measured via DigiGait. Then, mice were infected with TMEV or sham-infected (control). Mice were evaluated for neurological phenotypes twice a day for the first 14 d.p.i. and once a week thereafter until 90 d.p.i. The phenotype scores obtained throughout the 90 days were statistically associated with the pre-infection DigiGait measurements.

All mice were subjected to phenotyping and score assignment as previously described [31,40,58]. TMEV neurological phenotypes varied in presentation and severity over 90 d.p.i. This variation was partly attributed to differences in the immune response [40], lesion burden [58], and gene expression [47]. The 90 d.p.i. phenotype score included cumulative scores for “qualitative” phenotypes measured over 90 days. These phenotypes include paresis, paralysis, piloerection, kyphosis, seizures, limb clasping, and delayed righting reflex. Each phenotype was scored as “1” if present or “0” if not present during each observation. Infected mice were compared with strain- and sex-matched sham-infected mice to ensure that phenotype scores were not influenced by strain-specific idiosyncrasies. Observations occurred twice daily for the first 14 days p.i., during acute infection when the health of the mice may decline rapidly, then weekly until 90 d.p.i. The total number of “1” scores compared with the total number of observations were calculated to determine the frequency of each phenotype. The sum of all phenotype frequencies at 90 d.p.i. was the “90 d.p.i. phenotype score.” No data from sham-infected mice were considered for association with the 90 d.p.i. phenotype score, as the phenotype scores for uninfected mice were all approximately zero. The summarized methods are shown in Figure 2.

### 4.4. Statistical Analyses

Statistical analysis included 131 independent DigiGait variables, all measured prior to TMEV infection. Because different DigiGait parameters were assessed with different units/scales (such as cm^2^, percentages, etc.), the independent variables were standardized (i.e., mean of zero and variance of one), bringing all into the same scale for a more interpretable downstream analysis. Due to the response being skewed, a logarithm transformation was performed for downstream statistical analysis.

Statistical analyses were performed using R software and the “stepAIC” function for stepwise regression. The stepwise regression procedure involved both forward and backward selection in a procedure that started with the “intercept only” model and proceeded according to the optimal stopping criterion to choose the final model. Stepwise regression for each of the limbs was performed separately to select pre-infection DigiGait variables significantly associated with the 90 d.p.i. phenotype score.

To understand the effect of strain on the association between 90 d.p.i. phenotype score and the DigiGait variables, a linear mixed effect model was fitted using lmer package. The “r.squaredGLMM” function from the “MuMIn” package was applied to calculate a conditional and marginal coefficient of determination for the mixed-effect model.

GraphPad Prism version 9.4.1 for Mac (GraphPad Software, San Diego, CA, USA) was used to perform one-way ANOVA tests for associating baseline limb-specific DigiGait parameters with TMEV response group classifications (Resistant, Resilient, Susceptible), and two-way ANOVA tests for determining sex-specific differences among the Digigait parameters and the three TMEV response groups. All reported *p*-values were based on two-tailed statistical tests, with a significance level of 0.05.

### 4.5. Identification of Significant Quantitative Trait Loci (QTL)

Strain-averaged measurement values were used to identify QTL with statistically significant associations with DigiGait parameters, deemed to have a significant relationship with the 90 d.p.i. phenotype score. Measurement datasets were evaluated using gQTL software [65]; these datasets included measurements from all mice, female mice only, and male mice only. A total of 1000 permutations and *p* < 0.05 standards were applied to identify significant QTL. Next, strain-specific alleles for each QTL were identified using the Mouse Phenome Database [66] (assembly GRCm38). This search provided rsIDs and names of genes located nearest to the QTL SNPs, along with functional annotation for each SNP.

## 5. Conclusions

The severity of neurological symptoms following a viral infection can dictate the long-term quality of life. Symptom severity is, in part, influenced by pre-existing factors such as genetic background, which also affects immunological and physiological predispositions. In this study, baseline measurements of different aspects of gait, collected prior to infection, were statistically correlated with the severity of neurological symptoms during and after a viral infection. Mice with a shuffling and ambling gait prior to infection tended to be more severely affected after infection. Conversely, a more balanced and normal gait tended to predict a milder neurological outcome. Most intriguing, however, were the “resilient” mice which did not clear the viral infection but often had more divergent baseline gait profiles for parameters which had similar measurements in resistant and susceptible mice prior to infection.

## Figures and Tables

**Table 3:** Baseline DigiGait data were collected for 142 mice representing 22 mouse strains. Numbers of female and male mice are shown, along with their TMEV infection status. Not all strains were equally fecund and not all mice survived until 90 d.p.i., hence the differences in numbers of mice representing each sex and strain. 90 d.p.i. phenotype scores were calculated for each infected mouse included in the study, and the scores listed in the column to the far right represent the average of these scores for each strain. Some CC strains were previously identified as * resistant, ** resilient, or *** susceptible to TMEV-induced disease; these so-called “TMEV response categories” were defined in [47] and are summarized above. The SJL/J strain and CC recombinant strains CC002 × CC023, CC012 × CC032, and CC023 × CC002 were not included in the QTL analyses due to incompatibility with the gQTL program [63].

Strain	Infected F	Infected M	Sham F	Sham M	Total *n*	90 d.p.i. Phenotype Score
CC002 *	3	1	1	2	7	1.01
CC002 × CC023	0	0	2	2	4	0.14
CC005	0	1	1	1	3	0.14
CC011	1	2	1	1	5	0.43
CC012 × CC032	3	3	7	7	20	0.26
CC015 **	1	2	1	2	6	0.22
CC017	4	0	1	1	6	0.41
CC023 ***	6	8	2	2	18	2.31
CC023 × CC002	0	0	0	3	3	0.21
CC024	1	1	1	1	4	0.48
CC025	3	2	1	0	6	1.15
CC027 **	4	4	4	4	16	0.30
CC036 *	0	1	1	0	2	0.34
CC037 **	1	0	0	0	1	0.47
CC041	1	4	0	2	7	0.92
CC043 **	0	2	1	1	4	0.38
CC051 *	0	1	0	1	2	0.31
CC057	0	2	2	2	6	0.30
CC058	1	0	0	1	2	0.90
CC072	0	2	1	0	3	1.38
CC078	0	1	0	0	1	1.58
SJL/J	3	4	6	3	16	0.65

## Data Availability

All data supporting reported results can be found in Tables S1 and S2. Methods for gene expression data were published previously [47]; gene expression data relevant to this study are provided in Table S2.

## Supplementary Materials

The following supporting information can be downloaded at: https://www.mdpi.com/article/10.3390/ijms24032843/s1.
