# Supplementary material for: Baseline Gait and Motor Function Predict Long-Term Severity of Neurological Outcomes of Viral Infection
Source: Int J Mol Sci. 2023 Feb 2;24(3):2843. doi: 10.3390/ijms24032843 (PMC9917409; doi:10.3390/ijms24032843)

Figure S1: Influence of sex on relationships between TMEV response categories and pre-infection DigiGait measurements. *p<0.05, **p<0.01

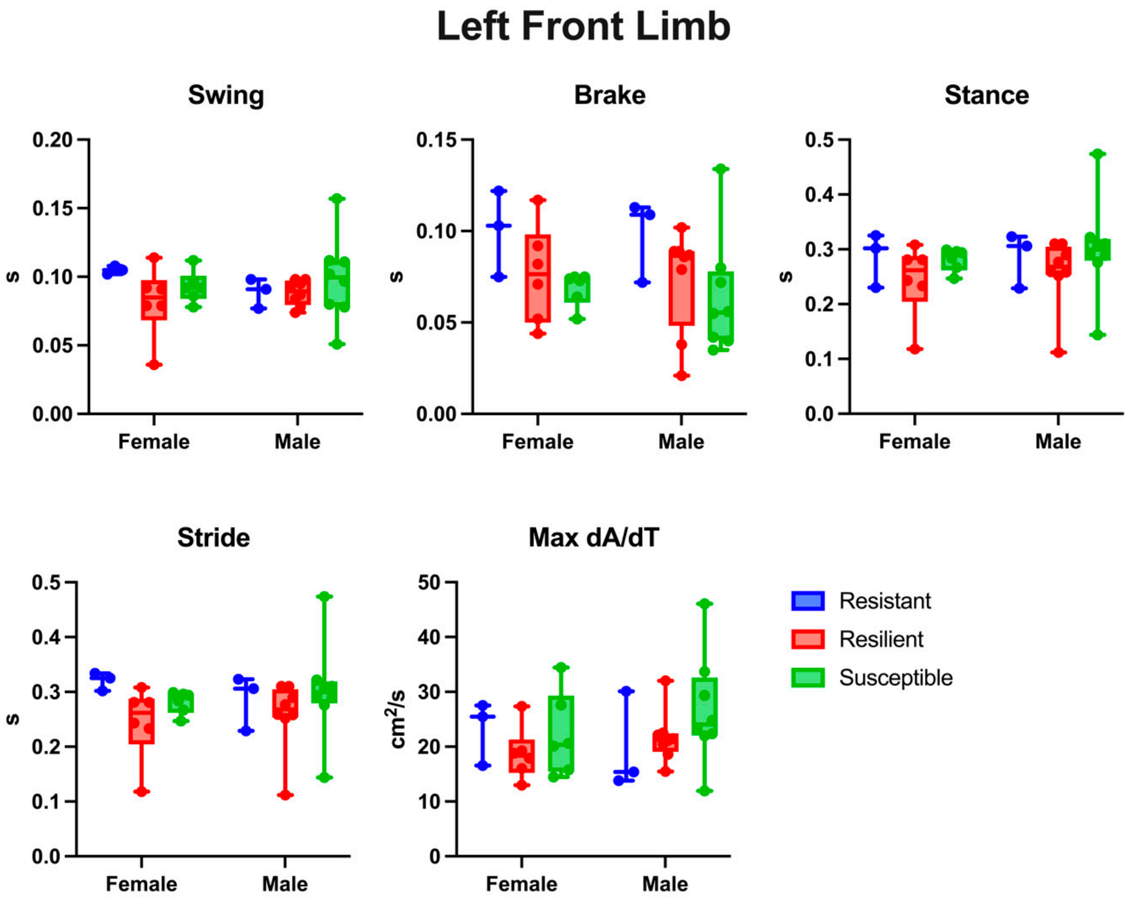

Left Hind Limb

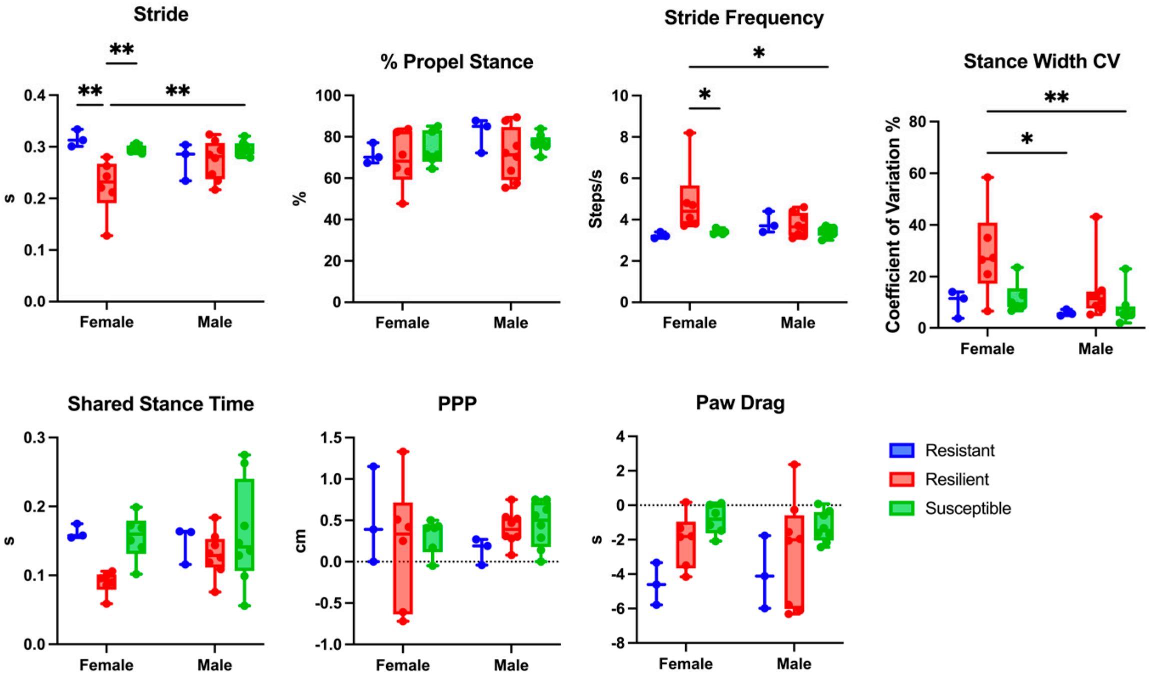

Right Front Limb

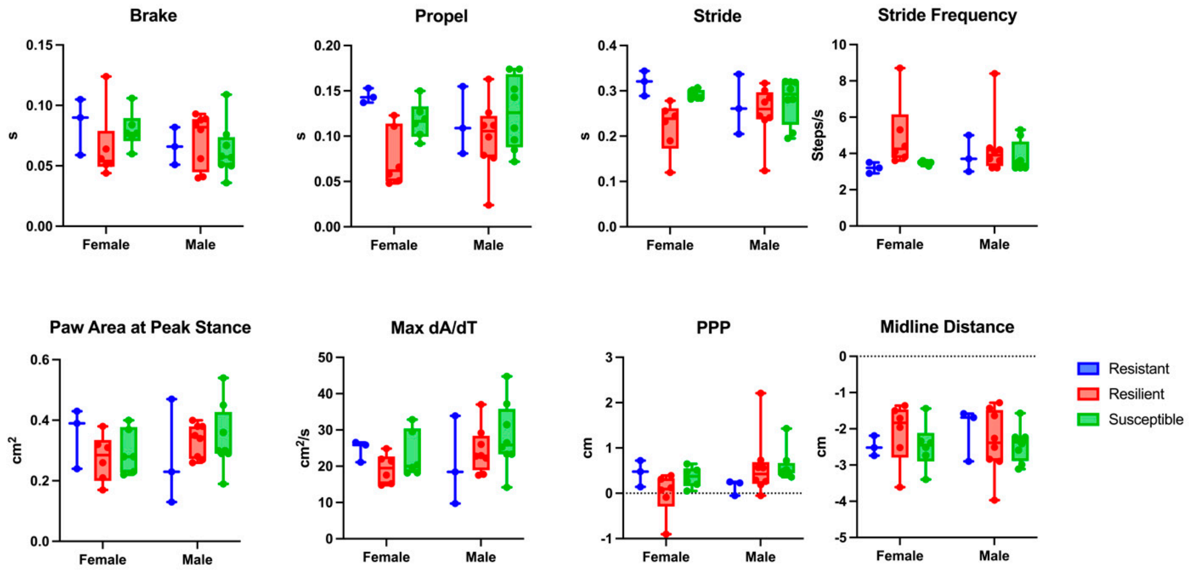

Right Hind Limb

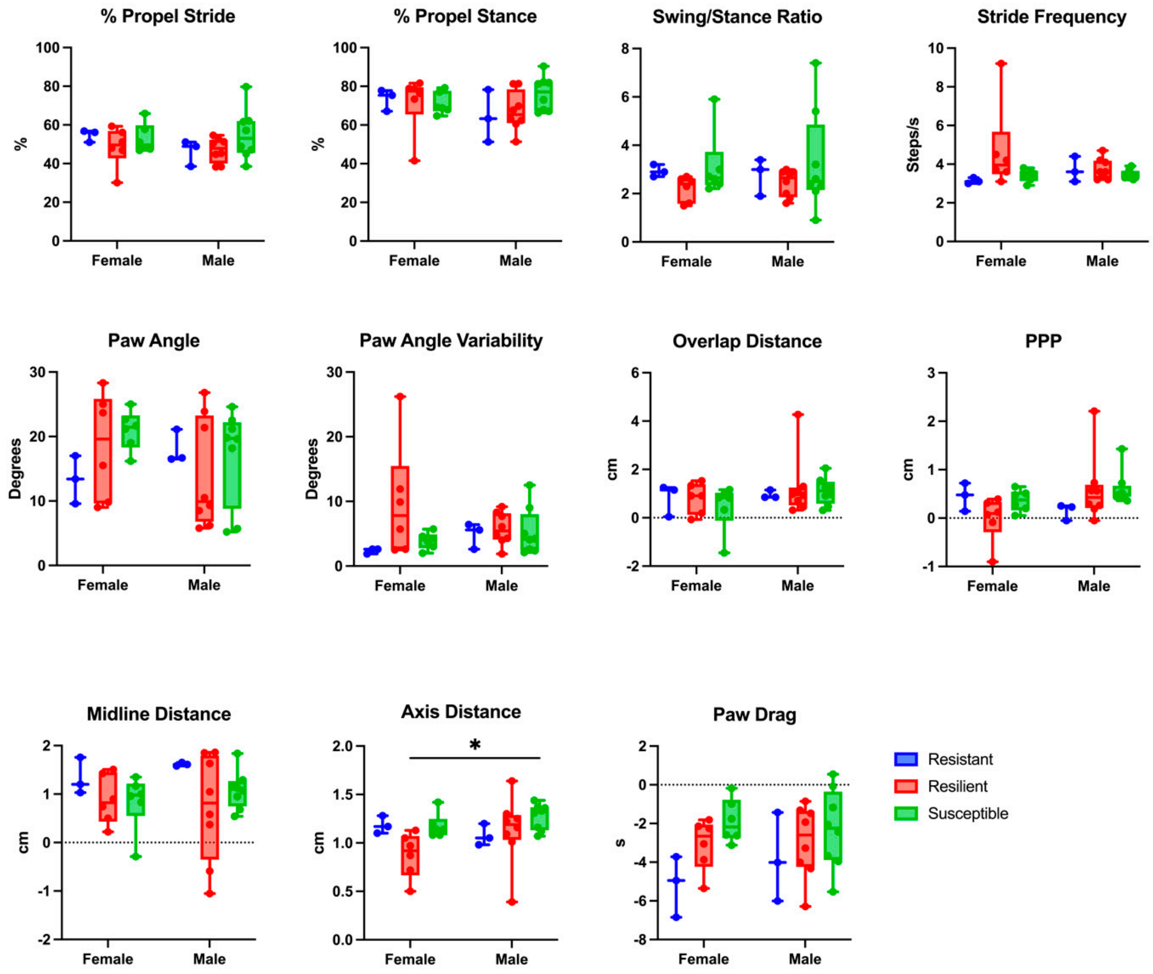

Supplement: Supplementary file 1 [file ijms-24-02843-s001.zip › Figure S1.pdf]
