# Supplementary material for: Baseline Gait and Motor Function Predict Long-Term Severity of Neurological Outcomes of Viral Infection
Source: Int J Mol Sci. 2023 Feb 2;24(3):2843. doi: 10.3390/ijms24032843 (PMC9917409; doi:10.3390/ijms24032843)

Figure S2: Manhattan plots and CC founder genotypes for significant QTL.

Significant QTL identified for both sexes (lowest dotted line indicates 85% significance, middle dotted line indicates 90% significance, and uppermost dotted line indicates 95% significance):

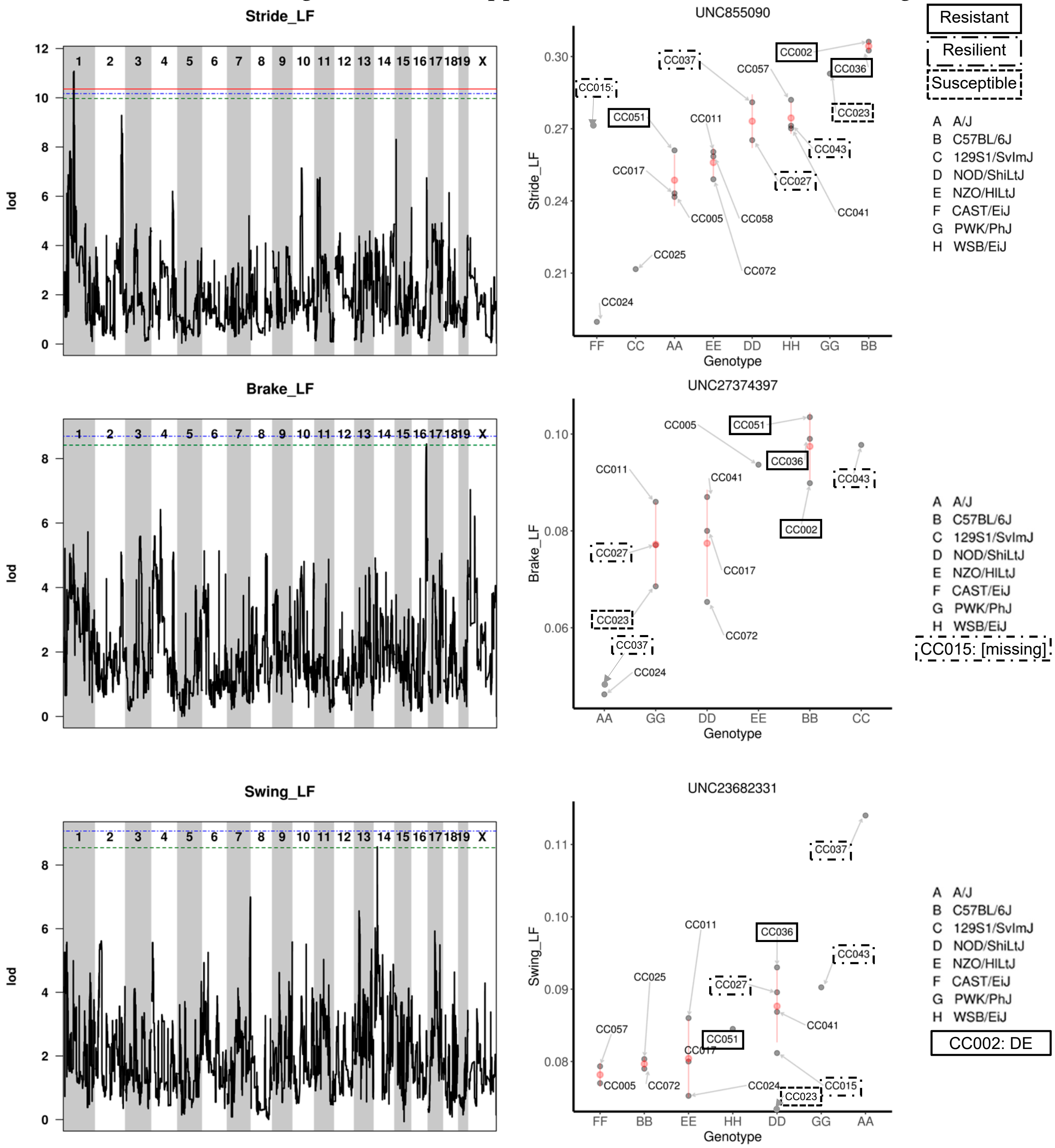

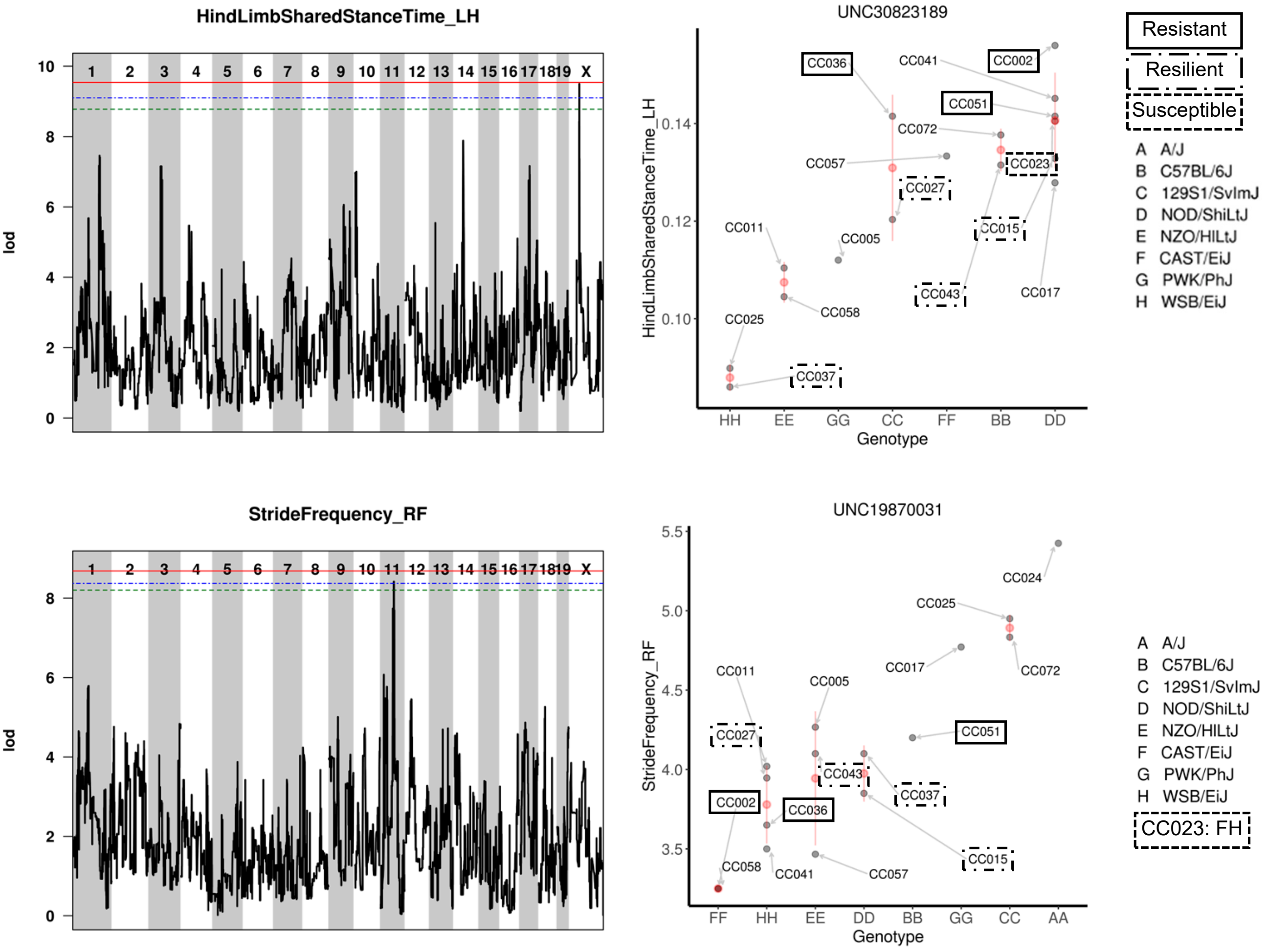

HindLimbSharedStanceTime_LH
lod
1 2 3 4 5 6 7 8 9 10 11 12 13 14 15 16 17 18 19 X
UNC30823189
HindLimbSharedStanceTime_LH
Genotype
HH EE GG CC FF BB DD
CC036
CC041
CC002
CC051
CC072
CC057
CC023
CC027
CC015
CC011
CC005
CC043
CC017
CC058
CC025
CC037
Resistant
Resilient
Susceptible
A A/J
B C57BL/6J
C 129S1/SvImJ
D NOD/ShiLtJ
E NZO/HILtJ
F CAST/EiJ
G PWK/PhJ
H WSB/EiJ
StrideFrequency_RF
lod
1 2 3 4 5 6 7 8 9 10 11 12 13 14 15 16 17 18 19 X
UNC19870031
StrideFrequency_RF
Genotype
FF HH EE DD BB GG CC AA
CC024
CC025
CC017
CC072
CC011
CC005
CC051
CC027
CC043
CC037
CC002
CC036
CC015
CC058
CC041
CC057
A A/J
B C57BL/6J
C 129S1/SvImJ
D NOD/ShiLtJ
E NZO/HILtJ
F CAST/EiJ
G PWK/PhJ
H WSB/EiJ
CC023: FH

Significant QTL identified for females only (lowest dotted line indicates 85% significance, middle dotted line indicates 90% significance, and uppermost dotted line indicates 95% significance):

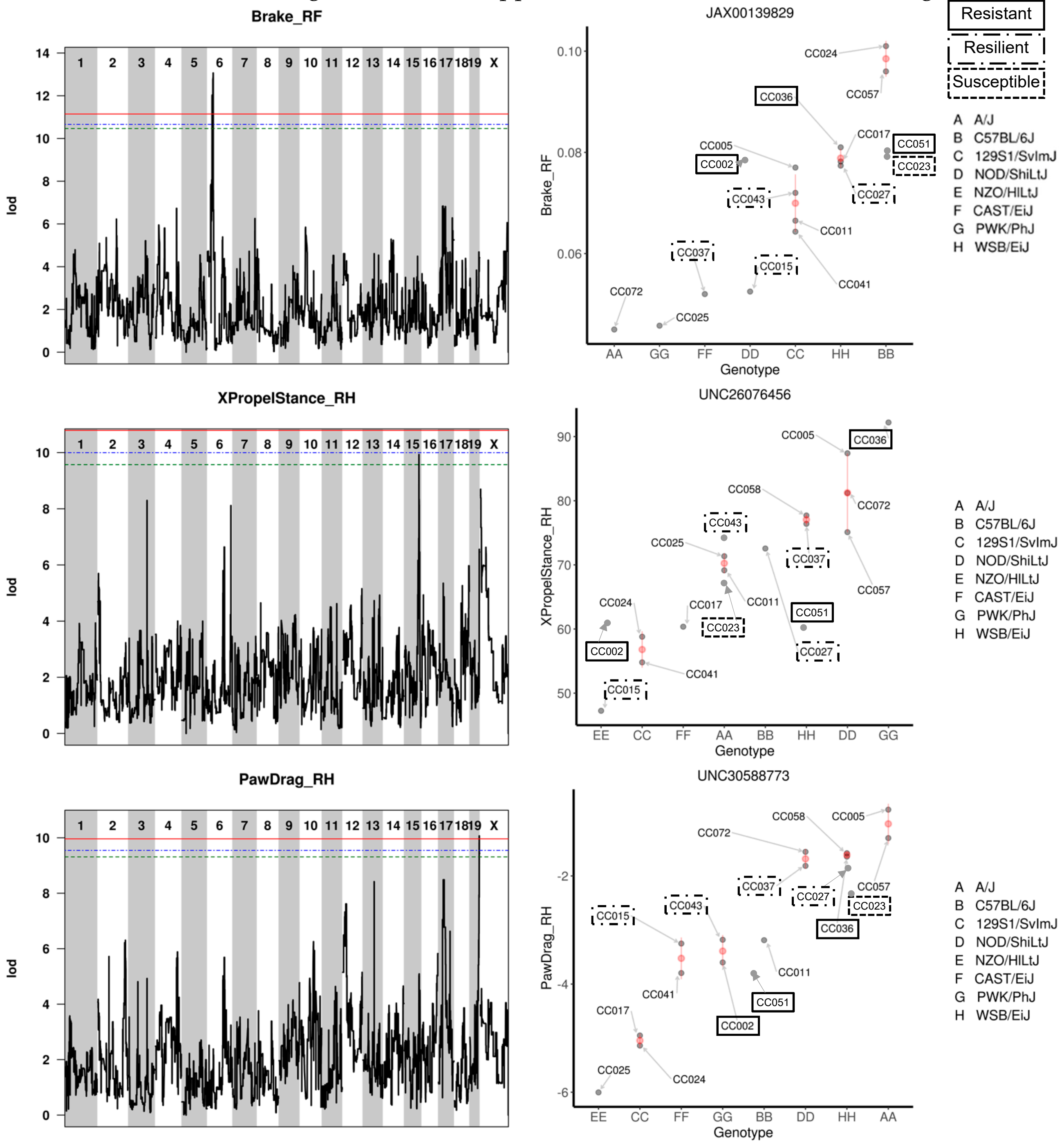

Significant QTL identified for males only (lowest dotted line indicates 85% significance, middle dotted line indicates 90% significance, and uppermost dotted line indicates 95% significance):

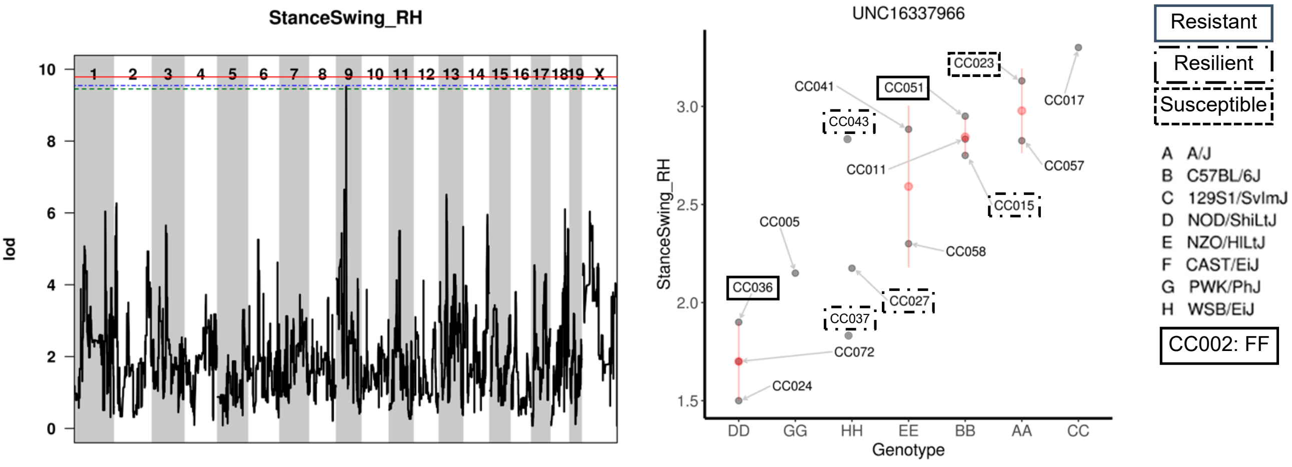

Supplement: Supplementary file 1 [file ijms-24-02843-s001.zip › Figure S2.pdf]
