# Supplementary material for: Baseline Gait and Motor Function Predict Long-Term Severity of Neurological Outcomes of Viral Infection
Source: Int J Mol Sci. 2023 Feb 2;24(3):2843. doi: 10.3390/ijms24032843 (PMC9917409; doi:10.3390/ijms24032843)

Figure S3: Founder genotypes are shown for QTL regions. All graphics are modified from data visualized by Collaborative Cross Viewer [49, 50]. Founder strain identities are distinguished by color, and strains identified as resistant, resilient, and susceptible are indicated by borders around the CC strain name, as indicated below each graphic.

Swing LF (both sexes): chr 14, 9.17-25.50 Mb

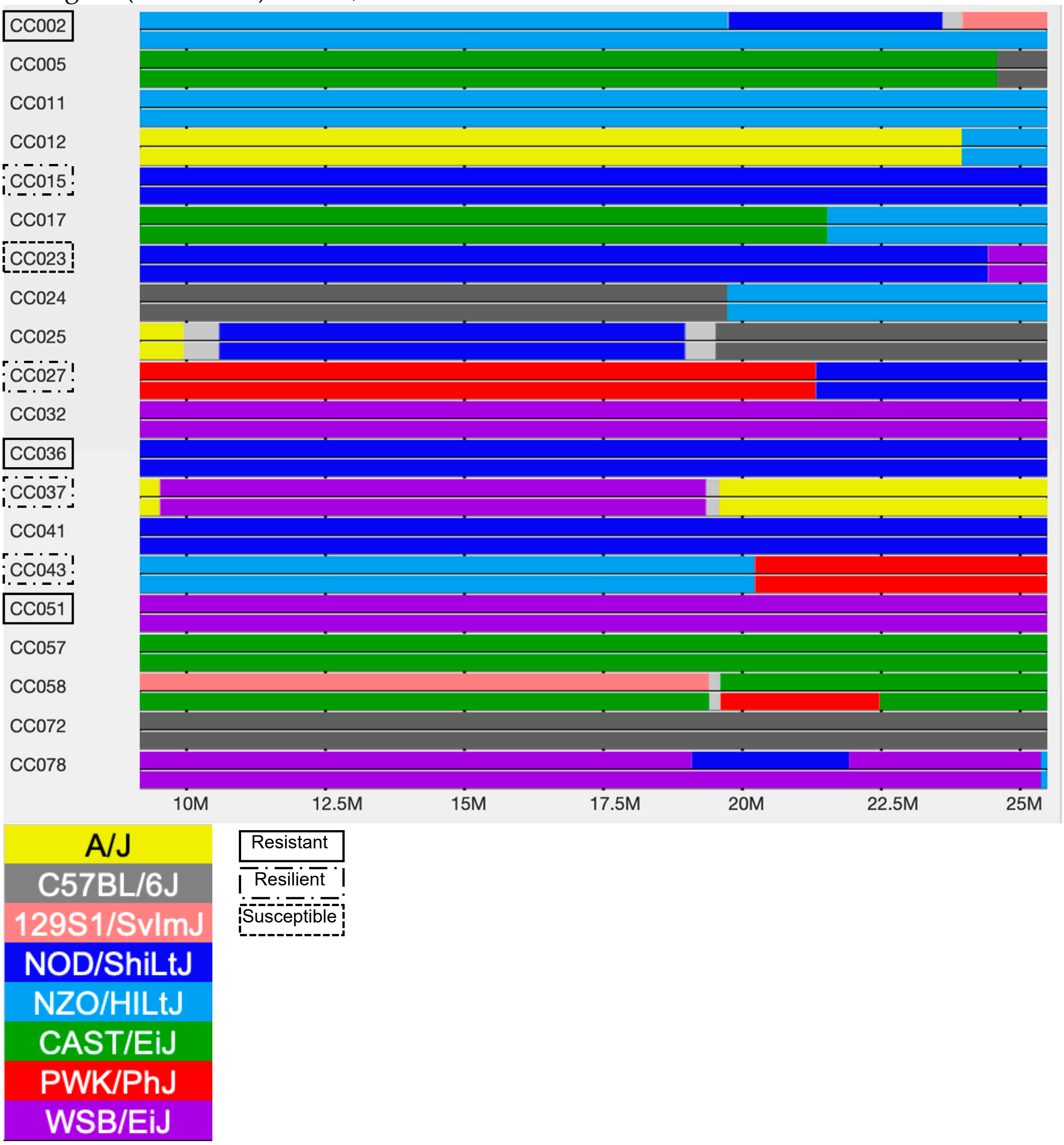

Brake LF (both sexes): chr 16, 86.46-90.56 Mb

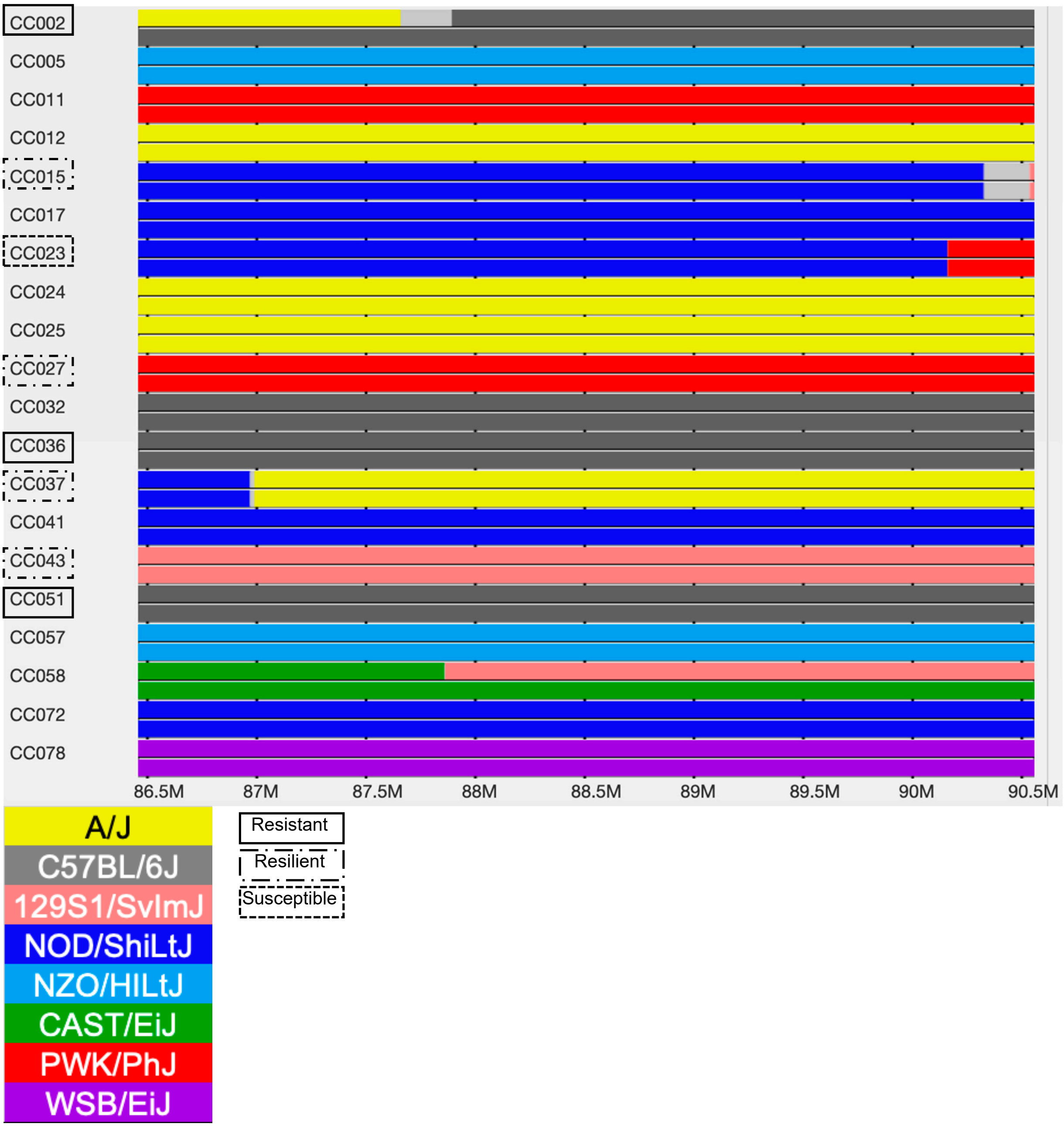

Stride LF (both sexes): chr 1, 64.57-67.96 Mb

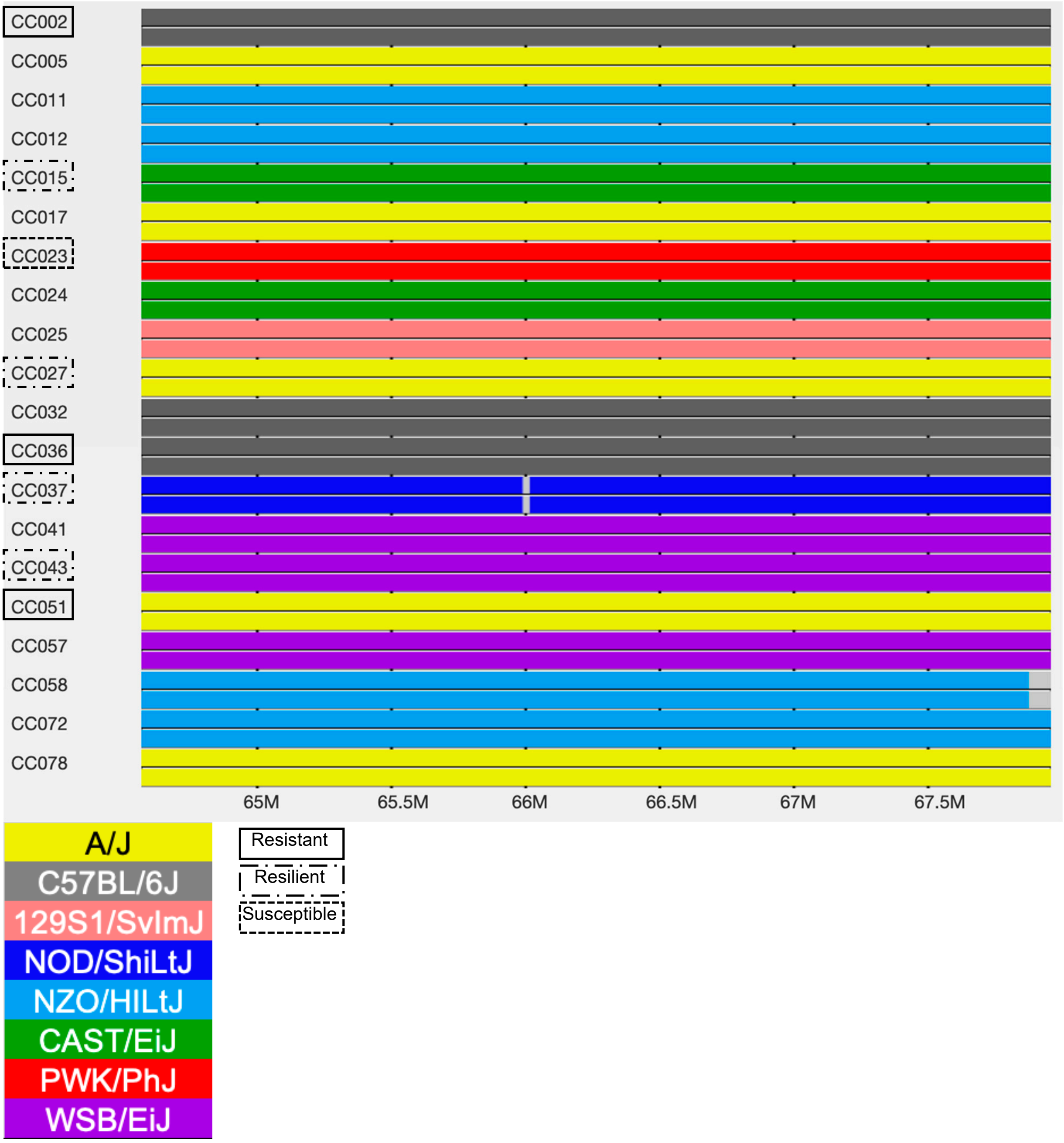

Hindlimb Shared Stance Time LH (both sexes): chr X, 50.67-51.84 Mb

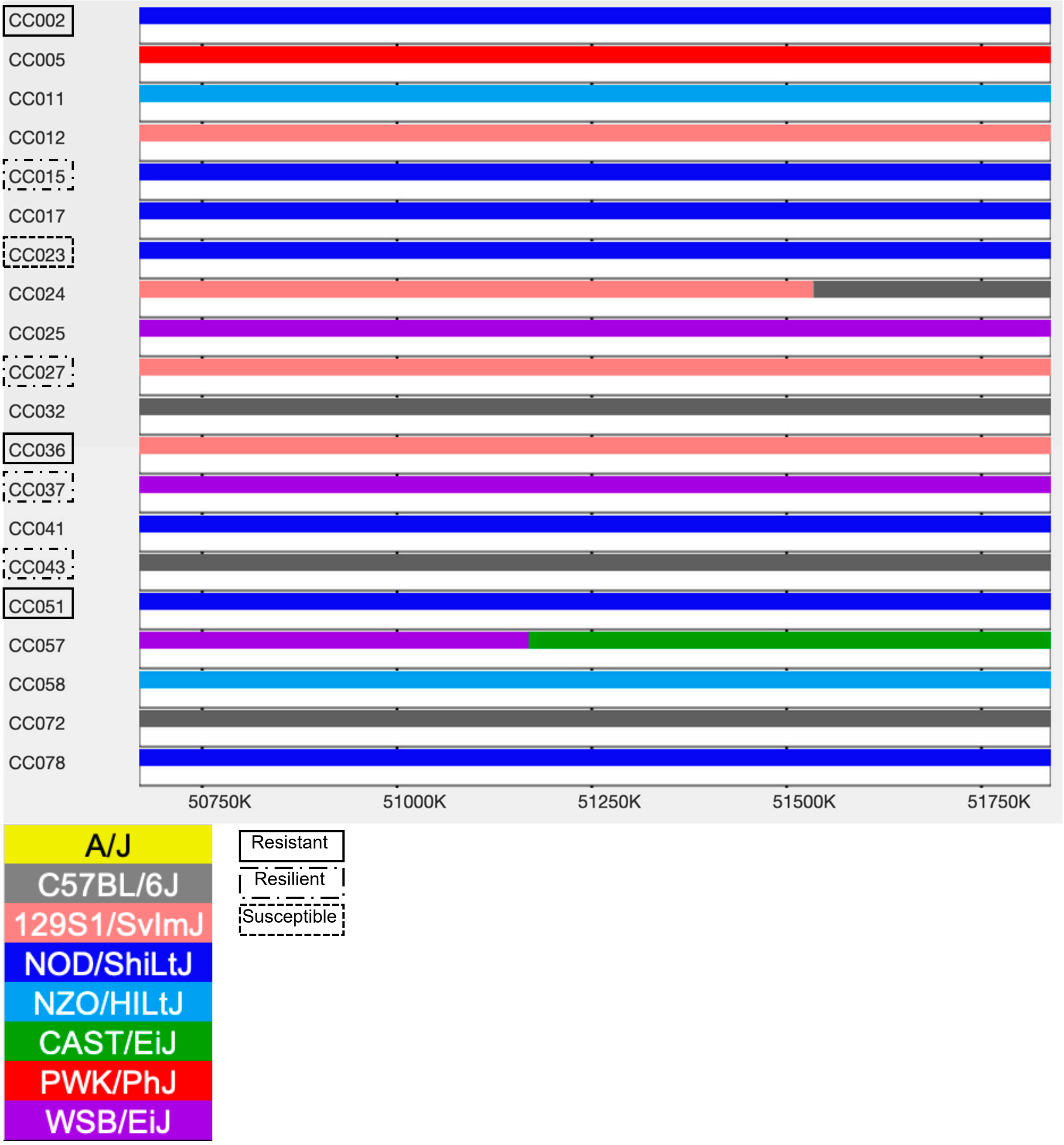

Brake RF (females only): chr 6, 34.85-36.99 Mb

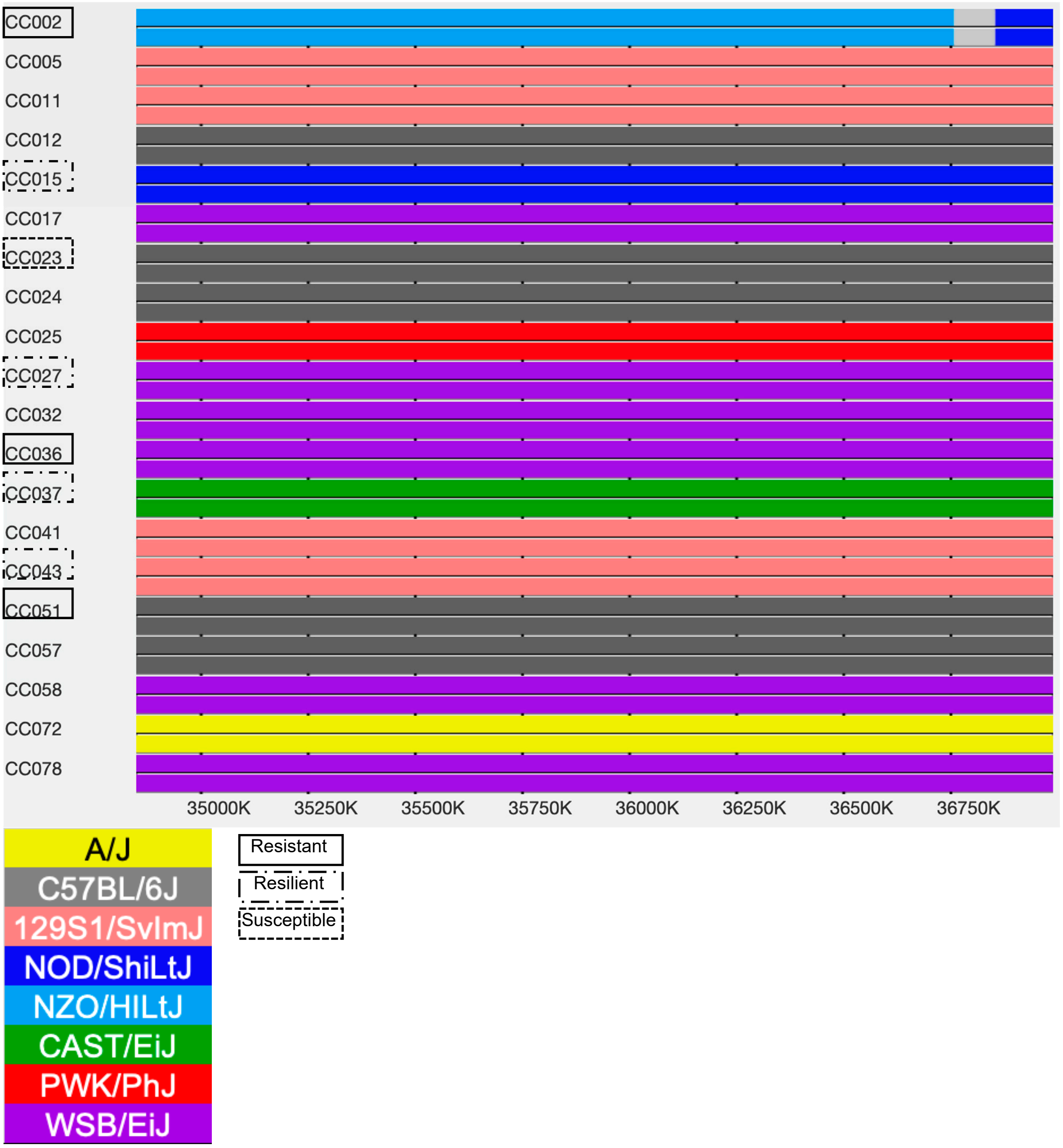

Stride Frequency RF (both sexes): chr 11, 64.57-67.96 Mb

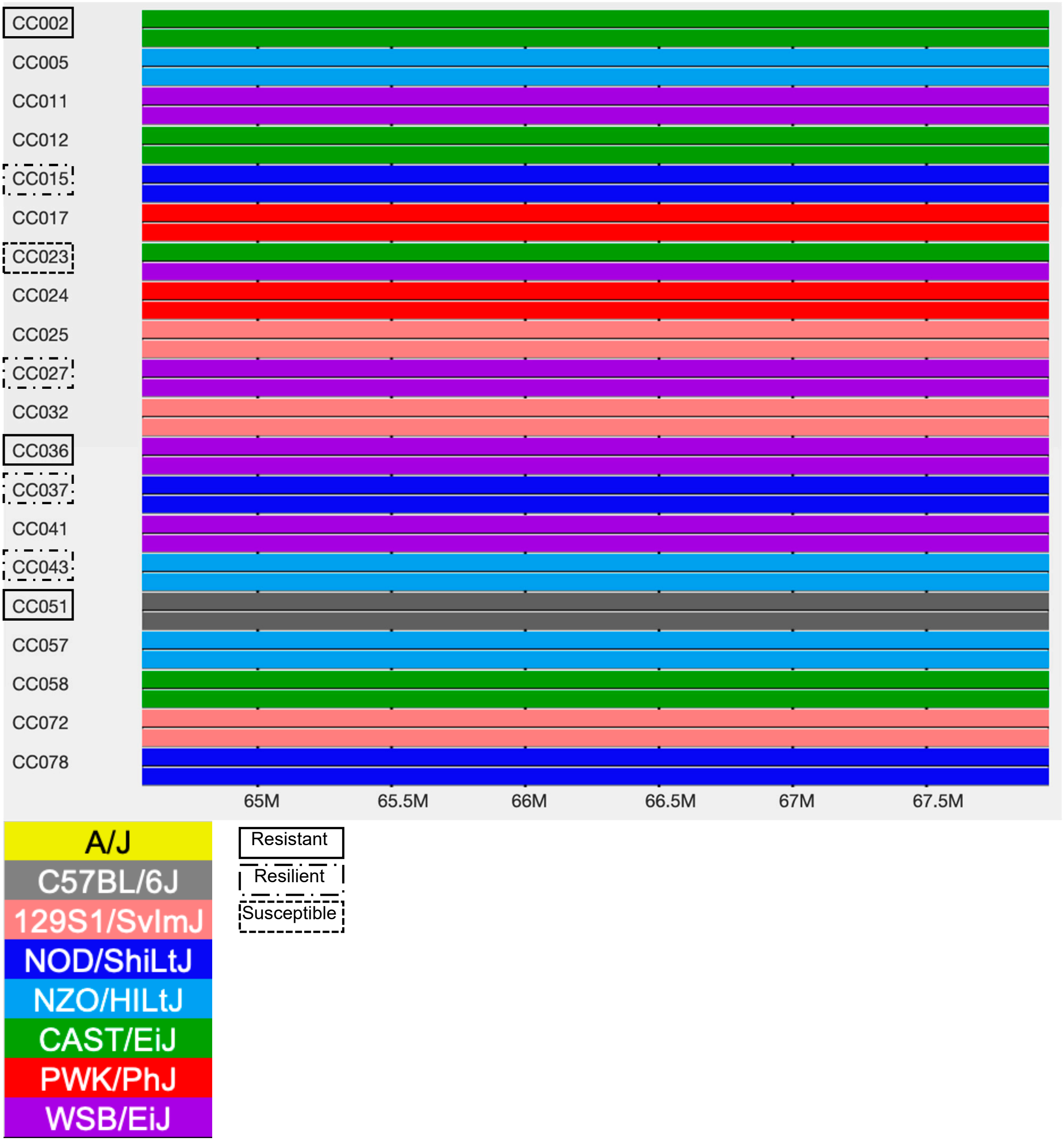

% Propel Stance RH (females only): chr 15, 89.59-91.33 Mb

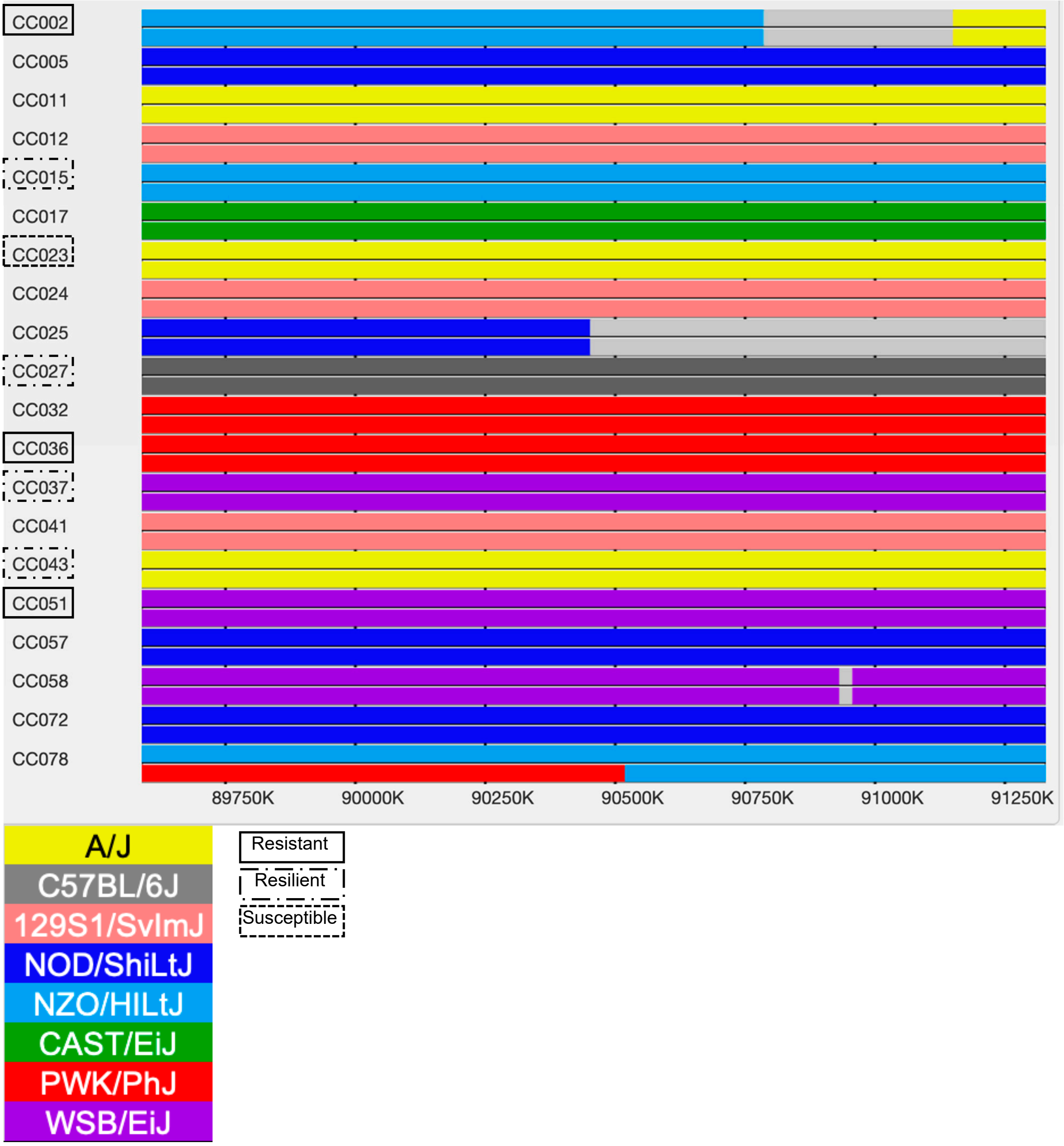

Stance/Swing RH (males only): chr 9, 49.70-52.20 Mb

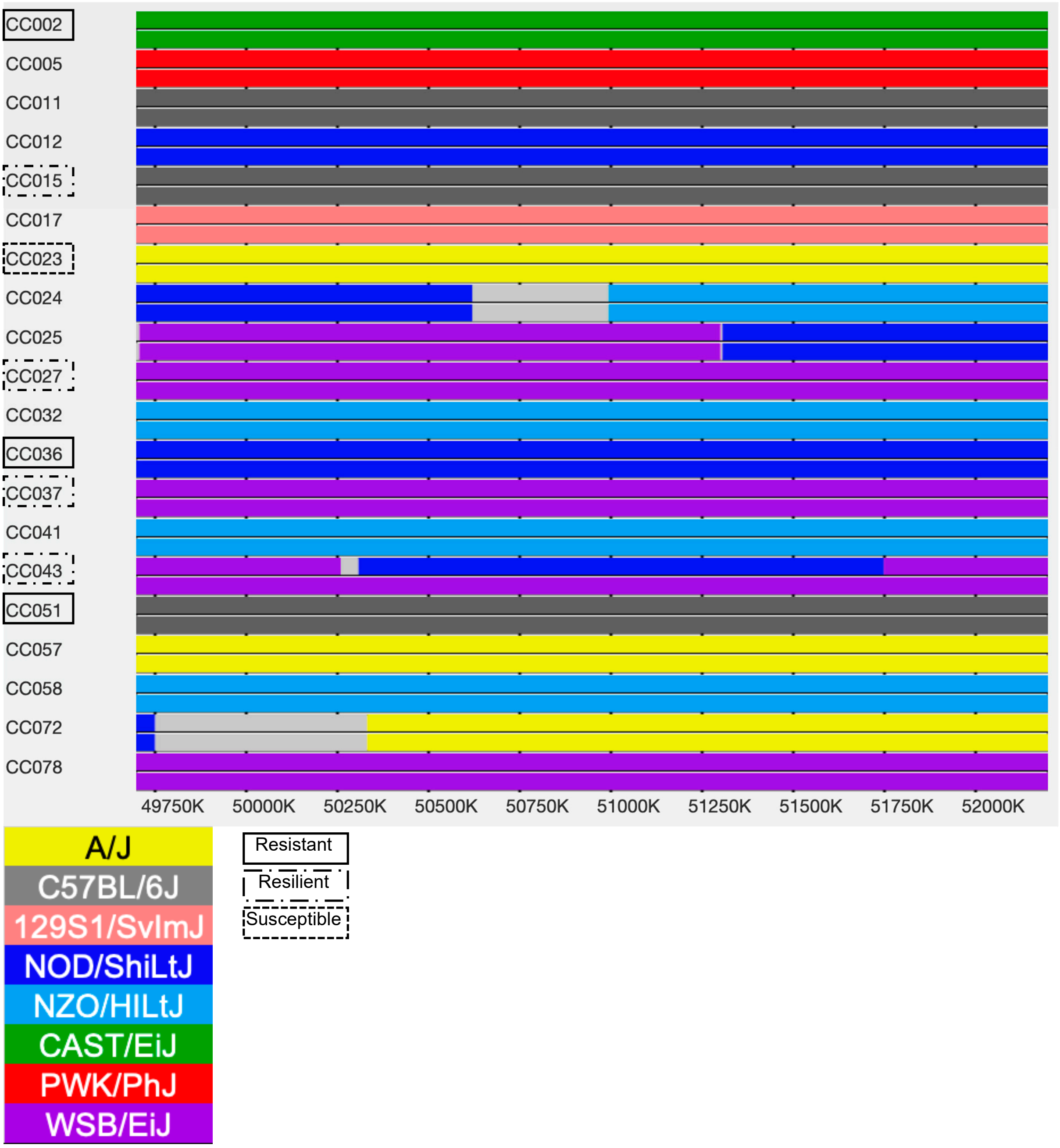

Paw Drag RH (females only): chr 19, 59.05-61.26 Mb

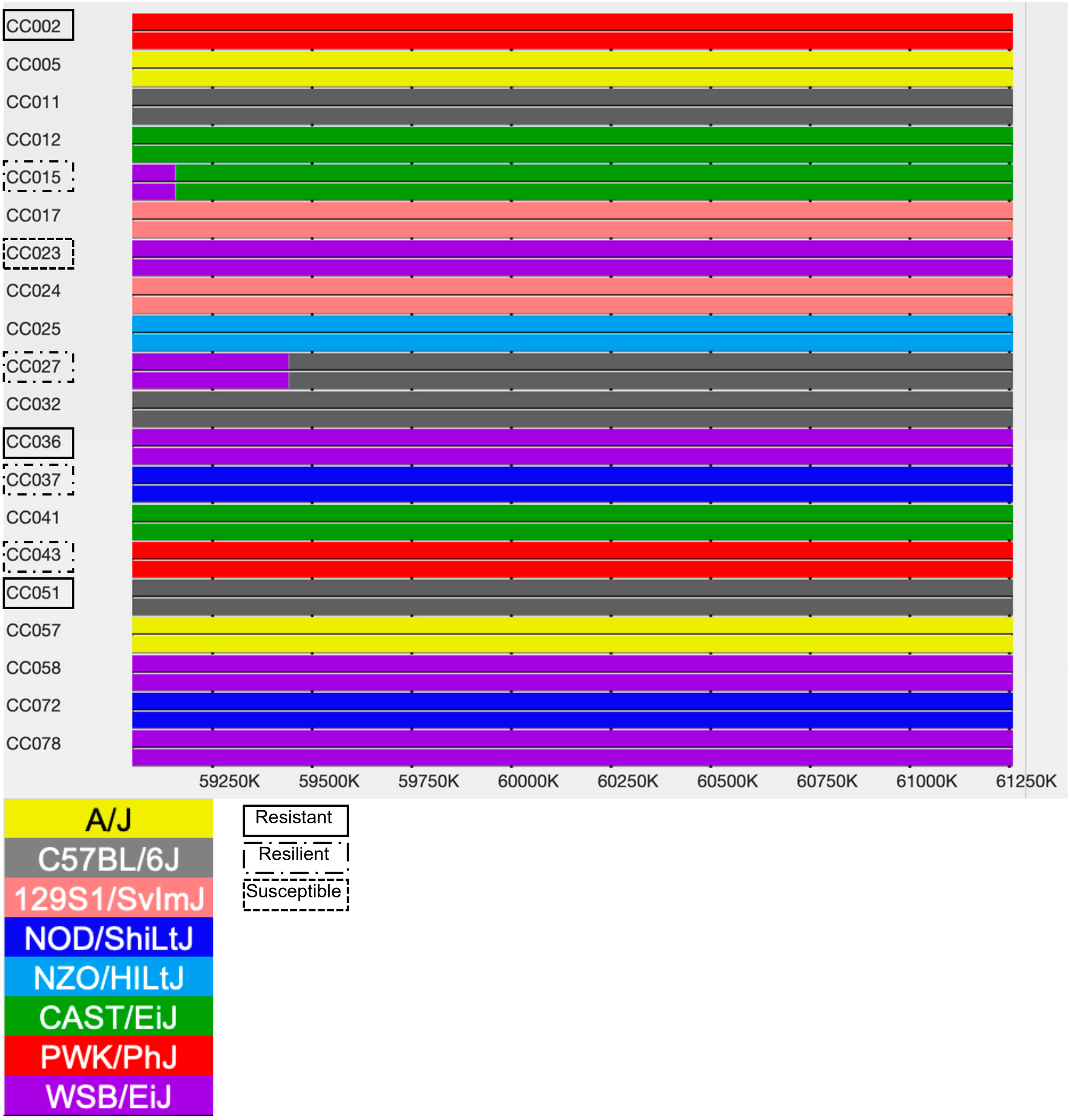

Supplement: Supplementary file 1 [file ijms-24-02843-s001.zip › Figure S3.pdf]
